# Characterization of the interaction between human *decidua parietalis* mesenchymal stem/stromal cells and natural killer cells

**DOI:** 10.1186/s13287-018-0844-y

**Published:** 2018-04-12

**Authors:** M. H. Abumaree, E. Bahattab, A. Alsadoun, A. Al Dosaimani, F. M. Abomaray, T. Khatlani, B. Kalionis, M. F. El-Muzaini, A. O. Alawad, A. S. AlAskar

**Affiliations:** 1 Stem Cells and Regenerative Medicine Department, King Abdullah International Medical Research Center, King Abdulaziz Medical City, Ministry of National Guard Health Affairs, P.O. Box 22490, Mail Code 1515, Riyadh, 11426 Saudi Arabia; 2 College of Science and Health Professions, King Saud Bin Abdulaziz University for Health Sciences, King Abdulaziz Medical City, Ministry of National Guard Health Affairs, P.O. Box 3660, Mail Code 3124, Riyadh, 11481 Saudi Arabia; 3 National Center for Stem Cell Technology, Life Sciences and Environment Research Institute, King Abdulaziz City for Science and Technology, P.O Box 6086, Riyadh, 11442 Saudi Arabia; 4 College of Medicine, King Saud Bin Abdulaziz University for Health Sciences, King Abdulaziz Medical City, Ministry of National Guard Health Affairs, P.O. Box 3660, Mail Code 3124, Riyadh, 11481 Saudi Arabia; 5 Department of Clinical Science, Intervention and Technology, Division of Obstetrics and Gynecology, Karolinska Institutet, 14186 Stockholm, Sweden; 6 Center for Hematology and Regenerative Medicine, Karolinska Institutet, 14186 Stockholm, Sweden; 7 Department of Maternal-Fetal Medicine Pregnancy Research Centre and University of Melbourne Department of Obstetrics and Gynaecology, Royal Women’s Hospital, Parkville, VIC 3052 Australia; 8 Department of Obstetrics and Gynaecology, King Abdulaziz Medical City, Ministry of National Guard Health Affairs, P.O. Box 3660, Mail Code 3124, Riyadh, 11481 Saudi Arabia; 9 College of Medicine, King Saud Bin Abdulaziz University for Health Sciences, King Abdulaziz Medical City, Ministry of National Guard Health Affairs, P.O. Box 3660, Mail Code 3124, Riyadh, 11481 Saudi Arabia; 10 Adult Hematology and Stem Cell Transplantation, King Abdulaziz Medical City, Ministry of National Guard Health Affairs, P.O. Box 22490, Mail Code 1515, Riyadh, 11426 Saudi Arabia

**Keywords:** *Decidua parietalis* mesenchymal stem/multipotent stromal cells, NK cells, Cytolytic activity, NK cell proliferation, Cancer, Inflammatory molecules

## Abstract

**Background:**

Human *decidua parietalis* mesenchymal stem/multipotent stromal cells (DPMSCs) have unique phenotypic and functional properties that make them promising candidates for cell-based therapy. Here, we investigated DPMSC interaction with natural killer (NK) cells, and the effects of this interaction on NK cell phenotypic characteristics and functional activities.

**Methods:**

DPMSCs isolated from the *decidua parietalis* of human fetal membranes were cultured with interleukin (IL)-2-activated and IL-2-unactivated NK cells isolated from healthy human peripheral blood. NK cell proliferation and cytolytic activities were then examined using functional assays. NK cell expression of receptors mediating the cytolytic activity against DPMSCs, and the mechanism underlying this effect on DPMSCs, were also examined using flow cytometry and light microscopy, respectively.

**Results:**

DPMSCs stimulated IL-2-induced proliferation of resting NK cells and the proliferation of activated NK cells. Moreover, IL-2-activated NK cells, but not freshly isolated NK cells, efficiently lysed DPMSCs. The induction of this NK cell cytolytic activity against DPMSCs was mediated by the activating NK cell receptors NKG2D, CD69, NKp30, and NKp44. However, DPMSCs showed a direct induction of NK cell cytolytic activity through CD69. We also found that DPMSCs expressed the ligands for these activating NK cell receptors including Nectin-2, ULBP-2, MICA, and MICB. Although DPMSCs expressed HLA class I molecules, they were susceptible to lysis by NK cells, suggesting that HLA class I antigens do not play a significant role in NK cell cytolytic action. In addition, DPMSCs did not inhibit NK cell cytolytic activity against cancer cells. Importantly, DPMSCs significantly increased NK expression of inflammatory molecules with anticancer activities.

**Conclusions:**

We conclude that DPMSCs have potential for therapeutic application in cancer therapy, but not in transplantation or immunological diseases.

## Background

Mesenchymal stem or stromal cells (MSCs) are adult cells with multiple differentiation potentials to form different tissues, such as adipose tissue, bone, and cartilage [1, 2]. MSCs can be isolated from many adult tissues such as the placenta and umbilical cord [3–8]. Previously, we reported the isolation and characterization of MSCs from the maternal side of the human placenta known as *decidua parietalis* (DPMSCs) [7, 9]. DPMSCs differentiate into the three characteristic mesenchymal lineages (adipocytes, chondrocytes, and osteocytes), and can proliferate and migrate in response to different stimuli [7, 9]. In addition, DPMSCs express many biological and immunological factors that are involved in important cellular functions including proliferation, differentiation, migration, immunomodulation, and angiogenesis [7]. These distinctive characteristics of DPMSCs make them an attractive candidate for cellular therapy.

It is well established that MSCs can modify the functions of several immune cells, such as lymphocytes (T and B cells) and antigen-presenting cells (macrophages and dendritic cells) [10–12]. In addition, MSC interaction with natural killer (NK) cells was also reported by a few studies [13–16]. It was shown that the coculture of MSCs isolated from human bone marrow (BMMSCs) and NK cells can result in MSC lysis [13–16]. However, the interaction between DPMSCs and NK cells is currently unknown.

NK cells are lymphocytes that are generated as progenitor cells from hematopoietic stem cells in bone marrow and which then appear as mature cells in the blood circulation [2]. They have specific immune functions to remove both virally infected cells and tumor cells [17]. A number of cell surface receptors known for their stimulatory and inhibitory functions are expressed by NK cells to mediate their cytolytic activity against target cells [17]. In addition, NK cell modulatory functions are also mediated by different cytokines (interferon (IFN)-γ, tumor necrosis factor (TNF)-α, interleukin (IL)-12 and IL-18) and their corresponding receptors [18, 19]. NK cells also express Toll-like receptors (TLRs) which mediate their modulatory functions [20, 21]. Moreover, NK cells secrete many cytokines and chemokines which can stimulate the functions of other immune cells [17].

NK cells produce several activating receptors (NKp30, NKp46, and NKp44), which mediate their cytolytic activity against target cells. These receptors are essential for the activation of NK cells following their interaction with their cognate ligands on target cells [22]. These receptors trigger NK cell cytolytic activities and their secretion of cytokines [23]. In addition, NK cells produce additional activating receptors (CD69, DNAM-1, and NKG2D) [24–29]. The ligands for DNAM-1 are PVR (poliovirus receptor) and Nectin-2, whereas NKG2D binds MICA/B (MHC class I chain-related gene A and B), as well as ULBPs (UL16 binding proteins) [24–29].

NK cells also express two inhibitory receptors, KIRs (killer immunoglobulin-like receptors) and CD94/NKG2A. KIRs are specific for human leukocyte antigen (HLA) class I [30] while CD94/NKG2A is specific for nonclassical MHC class 1 molecule (HLA-E) [31]. After binding their ligands on target cells, these inhibitory receptors inhibit NK cytolytic activity against target cells. In contrast, NK cell interaction with target cells lacking HLA molecules results in NK cell activation, which in turn induces the killing or lysis of target cells [32].

In this study, we determined the consequences of DPMSC interaction with NK cells. We found that DPMSCs stimulate proliferation of resting unactivated NK cells (NK cells induced to proliferate by IL-2) as well as activated NK cells (NK cells precultured with IL-2). We also found that IL-2-activated NK cells have a strong cytolytic activity against DPMSCs. In addition, DPMSCs did not interfere with NK cell cytolytic activity against cancer cells. Importantly, DPMSCs induced NK cell expression of inflammatory receptors including IL-18 receptors (IL-18Rα, IL-18Rβ) and TLR-7. These receptors mediate the antitumor activity of NK cells. We also found that the inhibition of DPMSC secretion of IL-12 reduced the expression of IL-18R in NK cells without affecting NK cell cytolytic activity against cancer cells. These results suggest that NK cells preconditioned by DPMSCs have anticancer therapeutic potential.

## Methods

### Ethics, collection of human placentae, and adult peripheral blood

The institutional research board (IRB) at King Abdulla International Medical Research Centre (KAIMRC), Saudi Arabia, approved this study. Placentae from uncomplicated human pregnancies (38–40 weeks of gestation) were collected after the signing of an informed consent form. The placentae were used immediately after delivery. Peripheral blood samples from healthy adult subjects were also obtained after the signing of an informed consent form.

### Isolation and culture of DPMSCs

MSCs were isolated from the *decidua parietalis* which lines the main cavity of the pregnant uterus and part of which attaches to the fetal membranes of the human term placenta. *Decidua parietalis* MSCs (DPMSCs) were isolated as we previously described [7, 9]. Briefly, the choriodecidua was manually separated from the amnion and washed intensively with phosphate-buffered saline (PBS; pH 7.4); 10 g of the tissue was extensively minced and the tissue suspension was then collected. After discarding the supernatant, red blood cell lysis was carried out using a lysing buffer (catalogue number sc-3621, Santa Cruz, California, USA) at room temperature for 45 min, and the tissue pellet was collected and incubated with 0.05% trypsin-EDTA (Life Technologies) and 270 unit/mL DNase I (Life Technologies) at 37 °C for 10 min. The digested tissue pellet was washed twice in Dulbecco’s modified Eagle’s medium nutrient mixture F-12 (DMEM-F12) containing 50% fetal bovine serum (FBS) for 10 min. Cells were pelleted and resuspended in DMEM-F12 medium (DPMSC complete growth medium) containing 20% MSC Certified FBS (MSCFBS, Life Technologies) and placed in a cell culture incubator (37 °C, 5% CO_2_ and 95% air). Cells were harvested with detachment solution (TrypLE express, Life Technologies) and then analyzed by flow cytometry using MSC and hematopoietic markers (Table 1) as previously published [33]. DPMSCs (passage 3) were evaluated for differentiation into adipocytes, osteocytes, and chondrocytes by culturing them in adipogenic (catalogue number 390415), osteogenic (catalogue number 390416) and chondrogenic (catalogue number 390417) media (R&D Systems, Abingdon, UK), respectively, as previously published [7]. Adipocytes, osteocytes, and chondrocytes were detected using Green Neutral Lipid Stain (LipidTOX™, Life Technologies), Alizarin Red S (Sodium alizarinsulfonate) stain, and Alcian Blue stain, respectively, as described previously [7]. DPMSCs (passage 3) of twenty placentae were used in subsequent experiments.

**Table 1:** Antibodies used in this study

MSC-positive markers	Hematopoietic markers	NK cell marker	NK cell activating receptors	NK cell inhibitory receptor	NK cell receptor ligands	Immune proteins
CD44	CD14	CD56	CD69	CD94/NKG2A	PVR (CD155)	IL-12Rβ1
CD90	CD19		CD226		Nectin-2 (CD112)	IL-18Rα
CD105	CD40		NKp30		ULBP-1	IL-18Rβ
CD146	CD45		NKp44		ULBP-2	IFN-γR1
CD166	CD80		NKp46		ULBP-3	IFN-γR2
HLA-ABC	CD83		NKpG2D (CD314)		MICA	TLR3
	CD86				MICB	TLR7
	HLA-DR				HLA-E	TLR9
						TNF-α

### Human NK cell isolation

Peripheral blood mononuclear cells (PBMNCs) were isolated from 10 normal healthy subjects using a Ficoll-Paque Leucosep™ density gradient solution (Greiner Bio-One, Kremsmünster, Austria). NK cells were isolated from PBMNCs using a human NK Cell Isolation Kit (catalogue number 130–092–657, Miltenyi Biotec, Bergisch Gladbach, Germany) as instructed by the manufacturer. Briefly, PBMNCs were resuspended in PBS solution (pH 7.2) containing 2 mM EDTA and 0.5% bovine serum albumin, and then the cells were incubated with biotin-antibody cocktail at 4 °C for 5 min. Following incubation of cells with microbead cocktail at 4 °C for 10 min, the cells were washed and resuspended in PBS buffer solution, and then loaded onto a column positioned in a magnetic separator (MACs; Miltenyi Biotec). Cells were then allowed to pass through and the effluent containing the unlabeled enriched NK cells was collected. The viability and purity of NK cells was evaluated byTrypan blue and anti-CD56 monoclonal antibody (R&D Systems) in flow cytometry, respectively. The viability and purity of NK cells used in this study were more than 95%. After isolation, NK cells were used either immediately or after 72-h activation with 100 U/mL IL-2 (R&D Systems) in RPMI 1640 medium containing 10% FBS, 2 mM l-glutamine, 50 μg/mL streptomycin, and 50 U/mL penicillin (NK culture medium).

### NK cell proliferation experiment

The proliferation of NK cells was measured with an MTS kit (a tetrazolium compound (3-(4,5-dimethylthiazol-2-yl)-5-(3-carboxymethoxyphenyl)-2-(4-sulfophenyl)-2H-tetrazolium, inner salt); catalogue no. G5421, Promega, Wisconsin, United States). Briefly, resting unactivated NK cells (NK cells induced to proliferate by treatment with 100 U/mL IL-2) or activated NK cells (NK cells precultured with 100 U/mL IL-2 for 72 h) as described previously [13] were cultured with or without DPMSCs at different ratios of NK:DPMSC (i.e., 1:1, 2.5:1, 5:1, 7.5:1, and 10:1). All cultures were performed in NK culture medium as described above. NK cell proliferation was then assessed after 72 h in culture using an MTS kit as instructed by the manufacturer. Briefly, cells were incubated in MTS solution for 4 h at 37 °C and then the absorbance at 490 nm was determined. Results from triplicate samples were presented as mean ± standard errors DPMSCs used in these proliferation experiments were initially treated with 25 μg/mL mitomycin C at 37 °C for 1 h to prevent their proliferation as previously described [34]. Different proliferation time points were also evaluated. Experiments were carried out in triplicate and repeated 10 times using 10 individual preparations of both NK cells and DPMSCs.

### NK cell cytolytic experiments

To determine the cytolytic potential of NK cells against DPMSCs, IL-2-unactivated NK cells and activated NK cells (NK cells precultured with 100 U/mL IL-2 for 72 h) were cultured with DPMSCs at different ratios of NK:DPMSC (i.e., 1:1, 5:1, 10:1, and 20:1). The activation of NK cells was performed as previously published [13]. Briefly, DPMSCs were cultured in DPMSC culture medium in six-well plates at 37 °C in a cell culture incubator. After 24 h, nonadherent DPMSCs were removed by washing with PBS, and adherent DPMSCs were cultured with IL-2 activated and unactivated NK cells at different ratios of NK:DPMSC (as indicated above) and then incubated at 37 °C. For monoclonal antibody blocking assays, NK cells were pretreated with antibodies specific to different NK cell activating and inhibitory receptors (Table 2) at 10 μg/mL (final concentration) at 4 °C for 30 min as previously described [13]. Following washing, NK cells were used in the cytolytic experiment at 10:1 NK:DPMSC ratio. Flow cytometry was used for the identification of DPMSC surface expression of ligands (Table 1) that are recognized by NK cell activating or inhibiting receptors. Controls were NK cells and DPMSCs, with each cell type cultured alone. Cells were visualized by light microscopy and photomicrographs were recorded. Cell lysis was assessed by determining the percentage of the remaining intact adherent cells per well. As described above, repeat experiments were performed using preparations of DPMSCs and NK cells.

**Table 2:** Monoclonal antibodies used in the blocking experiments

Monoclonal antibody	Isotype	Clone	Catalogue number
NKp30	IgG_2A_	210,847	MAB 1849
NKp44	IgG2a	253,415	MAB22491
NKp46	IgG2b	195,314	MAB 1850
CD69	IgG2a	298,633	MAB23591
CD226 (DNAM-1)	IgG1	102,511	MAB666
NKG2D (CD314)	IgG1	149,810	MAB139
CD94	IgG1	131,412	MAB1058

### Cytolytic activity of NK cells against tumor cells

To examine whether NK cell cytolytic activity was affected following their coculture with DPMSCs for 24 h, IL-2-activated NK cells cultured alone or cocultured with DPMSCs in the NK/DPMSC cytolytic experiment were harvested and then purified as described above. The viability and purity of NK cells were also assessed as described above. CD56^+^ NK cells with viability and purity of more than 95% were used against MCF-7 cells (breast cancer cells, ATCC, Manassas, VA, USA) at a ratio of 10:1 NK:MCF-7 cells. Cells were cultured in NK culture medium (described above) at 37 °C. Cells were then visualized by light microscopy and photomicrographs were recorded. Controls were NK cells or MCF-7 cells cultured alone. Different ratios of NK:MCF-7 were also evaluated, but a ratio of 10:1 NK:MCF-7 cells was chosen because it caused a complete lysis of MCF-7 cells. Assessment of cell lysis was performed as described above. Triplicate experiments were performed and repeated 10 times using NK cells isolated from 10 independent DPMSC/NK cytolytic assays and MC7 breast cancer cells.

### NK cell cytolytic activity against DPMSCs and tumor cells using xCELLigence real-time functional assay

To study the cytolytic activities of NK cells against DPMSCs or MCF-7, the real-time cell analyzer (xCELLigence RTCA-DP, Roche Diagnostics, Mannheim, Germany) was used as previously published [34, 35]. This real-time cell analyzer system records electrical impedance of cells on a conductive grid and this is reported as a cell index showing cellular events happening in real time [34]. For the NK/DPMSC cytolytic experiment, IL-2-unactivated or activated NK cells (as described above) were used against DPMSCs at different ratios (10:1 and 20:1 NK:DPMSC ratios). For the NK/MCF-7 cytolytic experiment, IL-2-activated NK cells cultured with DPMSCs in the NK/DPMSC cytolytic experiment (see above) were used against MCF-7 cells. After incubation of NK cells with DPMSCs (see above), NK cells were isolated and tested for viability and purity (as above) and then used against MCF-7 cells at a ratio 10:1 NK:MCF-7 cells. In both cytolytic experiments, 16-well E-culture plates (catalogue number 05469813001, Roche Diagnostics) were used and a background impedance was measured as instructed by the manufacturer. For each experiment, 2 × 10^4^ DPMSCs or MCF-7 cells, with or without NK cells at the indicated ratios, were seeded in NK culture medium in quadruplicate wells and left to equilibrate before data recording, as previously published [34]. After incubating the culture plates at 37 °C, the cell index of the DPMSC/NK or MCF-7/NK cultures was monitored in real time. The xCELLigence software (version 1.2.1) was used to analyze cell proliferation data (cell index) which were expressed as mean ± SD. The rate of cell growth was determined by calculating the linear regression of the slopes between two given time points. Five experiments were completed using DPMSCs (passage 3) and NK cells (*n* = 5 independent preparations each, with quadruplicate samples).

### NK cell expression of activating and inhibitory receptors and immune proteins

To examine the effects of DPMSCs on NK cell expression of activating and inhibitory receptors as well as immune proteins (Table 1) NK cells were harvested from the cytolytic assay and then purified using the NK Cell Isolation Kit and the magnetic cell separator as described above. Flow cytometry was then used to characterize NK cells.

### Mechanism mediating NK cell cytolytic activity against cancer cells

To examine whether the cytolytic activity of NK cells against cancer cells was mediated by IL-18 receptors (IL-18Rα and β), IL-2-activated NK cells were cocultured with DPMSCs at 10:1 NK:DPMSC ratio for 24 h as described above with or without IL-12 neutralizing monoclonal antibody (catalogue number MAB1510, R&D Systems) at a final concentration of 10 μg/mL. NK cells were then harvested as described above and their expression of IL-18Rα and β was characterized by flow cytometry. NK cytolytic activity against MCF-7 cells in the presence of DPMSC (10:1:1 NK:MCF-7:DPMSC ratio) and IL-12 neutralizing antibody was then determined using the xCELLigence system (as described above). Different concentrations of IL-12 neutralizing antibody were evaluated, but 10 μg/mL was chosen because it significantly inhibited IL-12 secretion as determined using a human IL-12/IL-23p40 enzyme-linked immunosorbent assay (ELISA) kit (catalogue number DP400, R&D Systems) as instructed by the manufacturer. Controls were NK cells or DPMSCs or MCF-7 cells cultured alone. Experiments were performed in triplicate and repeated three times using NK cells and DPMSCs prepared from three individual preparations of NK cells and DPMSCs.

### Flow cytometry

Cells (1 × 105) were stained with the antibodies listed in Table 1 for 30 min and then flow cytometry for cell surface and intracellular proteins was performed using a BD FACS CANTO II (Becton Dickinson) flow cytometer as previously described [36]. Negative controls were cells stained with fluorescein isothiocyanate (FITC)- or phycoerythrin (PE)-labeled mouse IgG isotype antibody.

### Statistical analysis

GraphPad Prism 5 was used to analyze data using nonparametric tests (Mann-Witney *U* and Kruskal-Wallis). Data were deemed statistically significant if *P* < 0.05.

## Results

### DPMSC isolation and characterization from *decidua parietalis* of human term fetal membranes

DPMSCs isolated from fetal membranes at passage 3 were > 95% positive for MSC markers and negative for hematopoietic markers (data not shown) as previously published by us [7]. In addition, DPMSCs differentiated into adipocytes, osteocytes, and chondrocytes in vitro (data not shown) as previously published by us [7]. Therefore, DPMSCs at passage 3 were used in all experiments in this study.

### DPMSCs stimulate NK cell proliferation

**Fig. 1:**
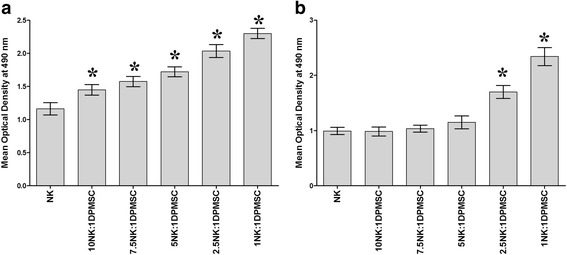
Effect of DPMSCs on NK cell proliferation. **a** Proliferation of natural killer (NK) cells was significantly increased in the presence of *decidua parietalis* mesenchymal stem/stromal cells (DPMSCs) as compared with untreated resting unactivated NK cells. **b** The proliferative response of NK cells in the presence of DPMSCs increased only at 2.5:1 and 1:1 NK:DPMSC ratios as compared with untreated IL-2-activated NK cells. Results of 10 representative experiments in which the proliferation of resting unactivated or IL-2-activated NK cells cultured for 72 h with or without mitomycin C-treated DPMSCs at different ratios of NK:DPMSC (10:1, 7.5:1, 5:1, 2.5:1, and 1:1) in the presence of 100 U/mL IL-2 was measured using MTS proliferation method. Experiments were carried out in triplicate and repeated 10 times using NK cells and DPMSCs prepared from the peripheral blood of 10 different healthy donors and 10 different fetal membranes, respectively. Bars represent standard errors. *P < 0.05

To examine whether DPMSCs can modify NK cell proliferation, DPMSCs were cultured with resting unactivated NK cells or activated NK cells. After 72-h culture, DPMSCs significantly increased the proliferation of unactivated NK cells as compared with untreated unactivated NK cells at all examined ratios (ranging from 10:1 to 1:1 NK:DPMSC; *P* < 0.05; Fig. 1a). In contrast, DPMSCs only increased the proliferation of activated NK cells at 2.5:1 and 1:1 NK:DPMSC ratios (*P* < 0.05; Fig. 1b).

### DPMSC surface expression of ligands bound by NK cell receptors

The possible interaction between NK cells and DPMSCs was examined by evaluating DPMSC expression of ligands known to bind to different NK cell receptors (activating and inhibiting receptors) using flow cytometry. The percentage of the expression of DNAM-1 and NKG2D (NK activating receptor ligands) and of HLA-E (NK inhibitory receptor ligands) in the DPMSCs is shown in Table 3. DPMSCs expressed Nectin-2 (ligand of DNAM-1) at high levels (63.26 ± 8.18%) and expressed low levels of NKG2D ligands including ULBP-2 (22.85 ± 5.37%), MICA (31.91 ± 5.67%), and MICB (22.40 ± 4.48%). In addition, DPMSCs expressed low levels of HLA-E (30.93 ± 8.36%) and high levels of HLA-ABC (95.60 ± 3.40%).

**Table 3:** Percentage of DPMSC expressing surface ligands of natural killer (NK) activating receptors (DNAM-1 and NKG2D) and the ligands of NK inhibiting receptor (HLA-E)

DNAM-1 ligands		NKG2D ligands					CD94/NKG2 ligand	HLA-ABC
PVR (CD155)	Nectin-2 (CD112)	ULBP-1	ULBP-2	ULBP-3	MICA	MICB	HLA-E	
1.00% ± 0.18%	63.26% ± 8.18%	7.95% ± 1.33%	22.85% ± 5.37%	5.63% ± .0.68%	31.91% ± 5.67%	22.40% ± 4.48%	30.93% ± 8.36%	95.60% ± 3.40%

Experiments were repeated 10 times using DPMSCs isolated from 10 different fetal membranes

### DPMSC lysis by NK cells

**Fig. 2:**
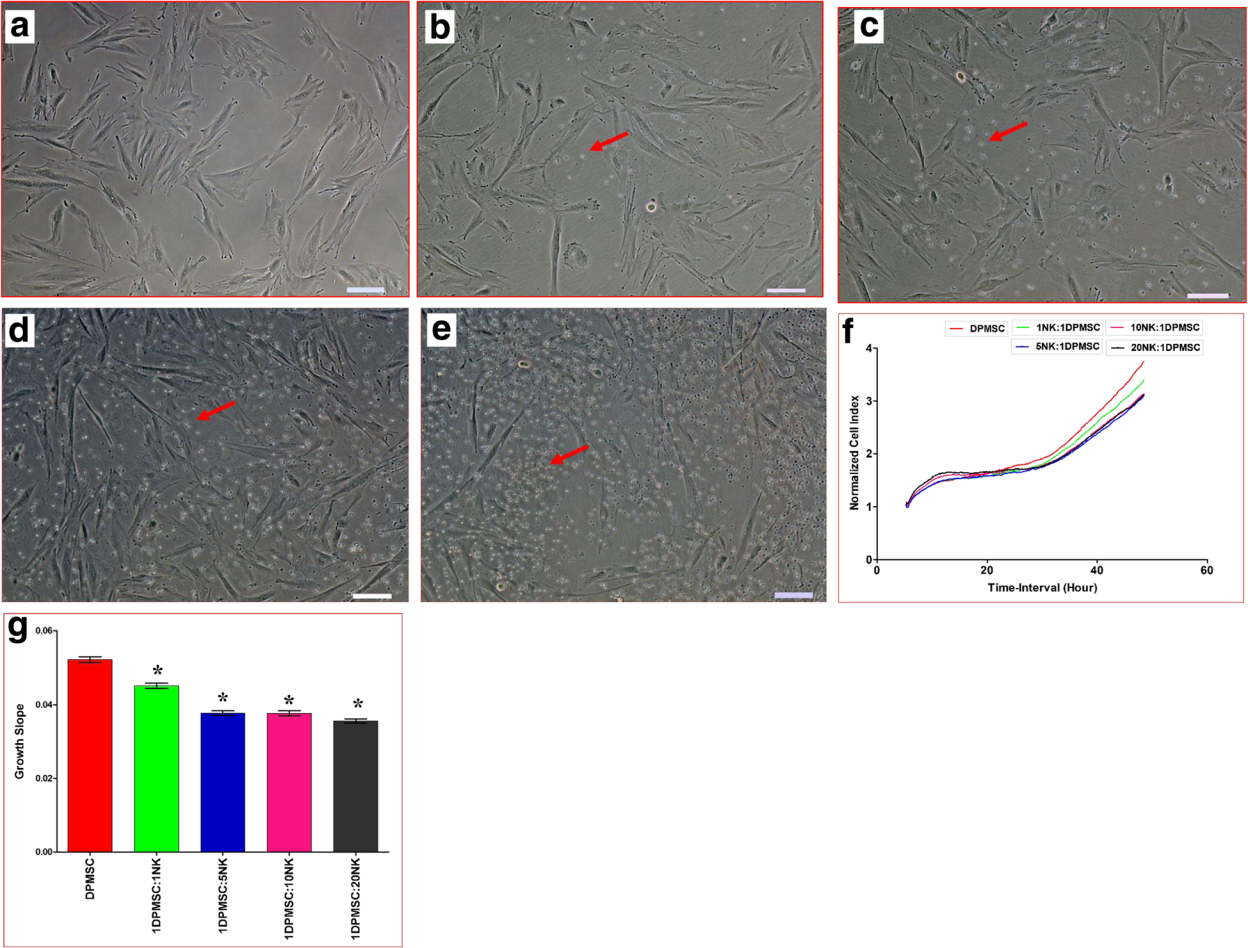
Natural killer (NK) cell interaction with *decidua parietalis* mesenchymal stem/stromal cells (DPMSCs) evaluated by culturing IL-2-unactivated NK cells with DPMSCs at different DPMSC:NK ratios for 24 h, and NK cell cytolytic activity against DPMSCs then assessed using microscopic examination and the xCELLigence real-time cell analyzer. Representative phase contrast microscopic images show untreated DPMSCs (control) with a spindle-like morphology (**a**), and nonlysed DPMSCs cultured with IL-2-unactivated NK cells (arrows) at ratios of 1:1 (**b**), 1:5 (**c**), 1:10 (**d**), and 1:20 (**e**) DPMSC:NK. The results of the xCELLigence showed that after 50 h of culture, IL-2-unactivated NK cells did not lyse DPMSCs as shown by the cell index (**f**) and significantly decreased DPMSC proliferation (growth slope) (**g**) at all examined NK:DPMSC ratios. Experiments were carried out in triplicate and repeated 10 times using NK cells and DPMSCs prepared from the peripheral blood of 10 different healthy donors and 10 different fetal membranes, respectively. Scale bars = 50 μm. *P < 0.05

**Fig. 3:**
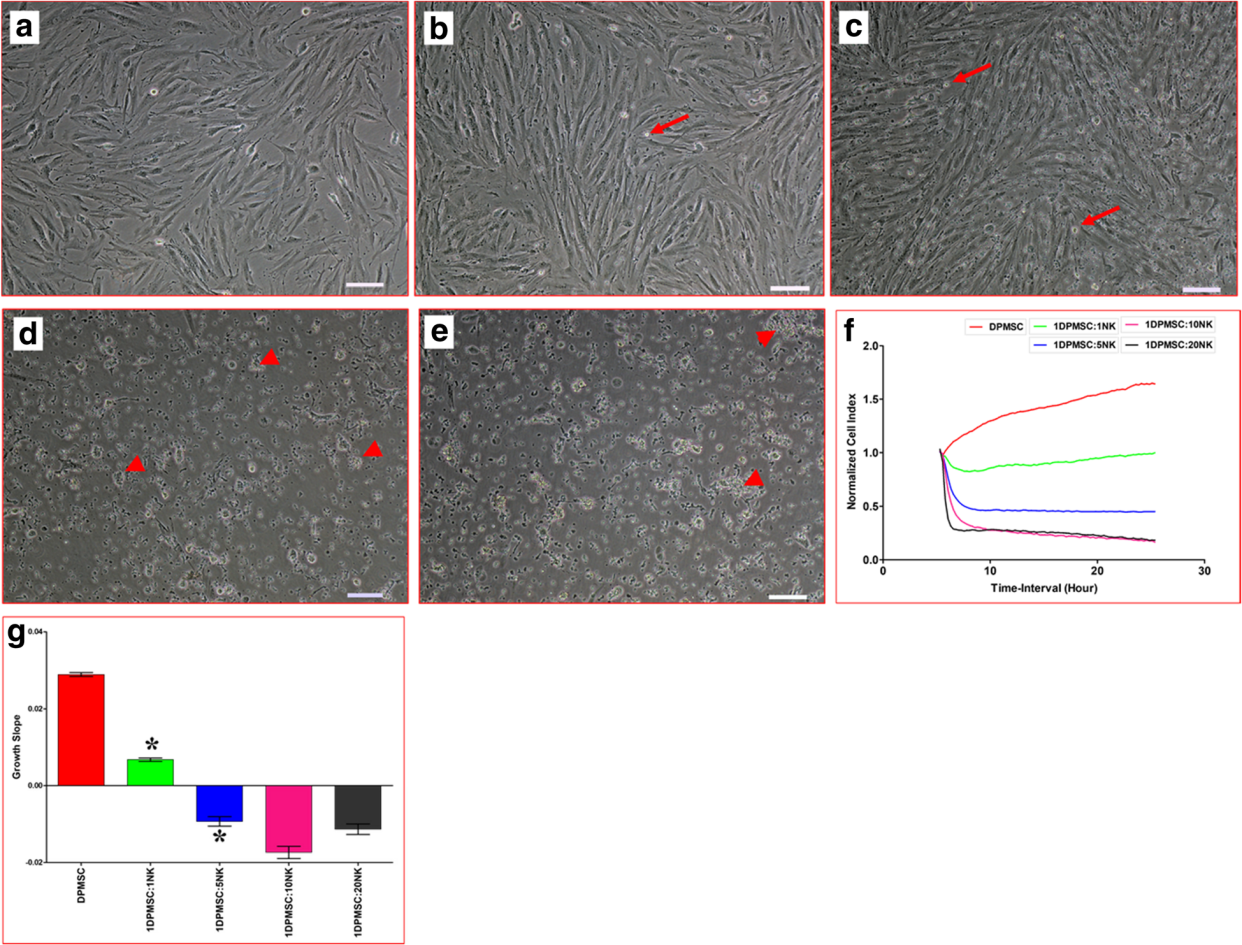
Natural killer (NK) cell interaction with *decidua parietalis* mesenchymal stem/stromal cells (DPMSCs) evaluated by culturing IL-2-activated NK cells with DPMSCs at different NK:DPMSC ratios for 24 h, and NK cytolytic activity against DPMSCs then assessed using microscopic examination and xCELLigence real-time cell analyzer. Representative phase contrast microscopic images show untreated DPMSCs (control) with typical spindle-like morphology (**a**), nonlysed DPMSCs cultured with IL-2-activated NK cells (arrows) at ratios of 1:1 (**b**) and 1:5 (**c**) DPMSC:NK, and lysed DPMSCs cultured with IL-2-activated NK cells (arrows) at ratios of 1:10 (**d**) and 1:20 (**e**) DPMSC:NK. The lysis of DPMSCs was evident as there was no sign of intact adherent DPMSCs, cells were ruptured, and cellular debris was obvious in suspension (arrowheads). The results of the xCELLigence showed that after 24 h culture IL-2-stimulated NK cells did not lyse DPMSCs at ratios of 1:1 and 1:5 DPMSC:NK, but DPMSCs were lysed at 1:10 and 1:20 DPMSC:NK as shown by the cell index (**f**), which showed the cell adhesion and proliferation of intact cells was reduced almost to zero, and growth slope (**g**). At a ratio of 1:1 and 5:1 NK:DPMSC, DPMSC proliferation significantly decreased (**f** and **g**). Experiments were carried out in triplicate and repeated 10 times using NK cells and DPMSCs prepared from the peripheral blood of 10 different healthy donors and 10 different fetal membranes, respectively. Scale bars = 50 μm. *P < 0.05

DPMSCs express ligands that are recognized by activating NK receptors, which suggest that DPMSCs could be lysed by NK cells. Therefore, we examined the ability of IL-2-unactivated and activated NK cells to lyse DPMSCs. As previously reported [13, 16], we found that IL-2-unactivated NK cells could not lyse DPMSCs (Fig. 2). However, NK cells significantly decreased DPMSC proliferation (*P* < 0.05) at all examined ratios (1:1, 1:5, 1:10, and 1:20 DPMSC:NK) as shown by the cell index (Fig. 2f) and slope of growth (Fig. 2g). In contrast, following the activation of NK cells with IL-2 for 72 h, NK cells significantly lysed DPMSCs, but at high NK ratios (10:1 and 20:1 NK:DPMSC ratios) within 24 h culture. At these ratios, NK cells completely lysed DPMSCs (with no sign of intact adherent DPMSCs, ruptured cells, and obvious cellular debris in suspension within 24 h; Fig. 3d, e). This NK cell cytolytic activity against DPMSCs was also confirmed by the xCELLigence real-time cell analyzer. The cell index which shows cell adhesion and proliferation of intact cells was reduced almost to zero for DPMSCs cocultured with NK cells (Fig. 3e and f). However, at lower NK ratios (1:1 and 5:1 NK:DPMSC), NK cells could not lyse DPMSCs at the time point (24 h) examined in this study, but significantly inhibited DPMSC growth as shown by the cell index (Fig. 3f) and slope of growth (Fig. 3g).

### Examination of the molecular interactions underlying DPMSC lysis

**Fig. 4:**
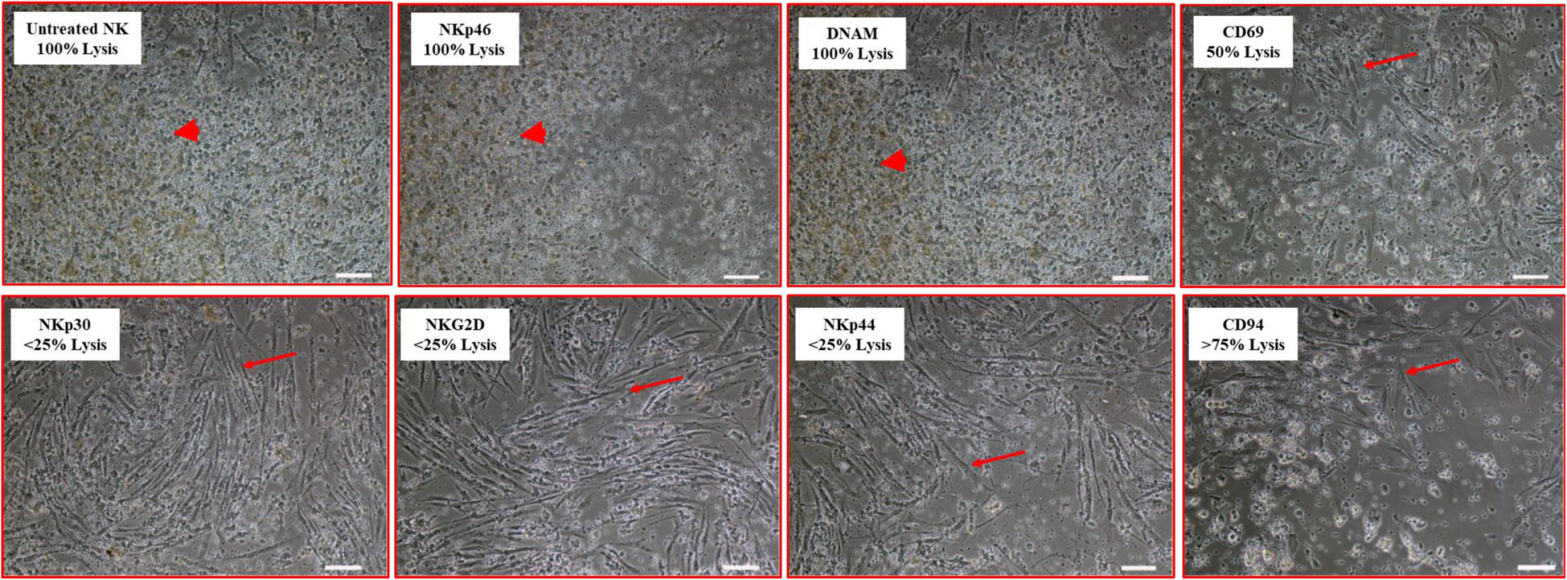
Antibody-mediated blocking experiments showing natural killer (NK) cell activating and inhibiting receptors mediating DPMSC lysis. NK cells were preincubated with antibodies specific to the indicated NK cell activating and inhibiting receptors; NK cells were then cocultured with DPMSCs at a 10:1 NK:DPMSC ratio in a cytolytic assay. DPMSC lysis by NK cells was evaluated using microscopic examination within 24 h. Representative phase-contrast microscopic images show complete lysis of DPMSCs (no sign of intact adherent DPMSCs, cells were ruptured, and cellular debris was obvious in suspension (arrowheads) by untreated NK cells (NK cells were initially activated with 100 U/ml IL-2 for 72 h), by NKp46, by DNAM, and moderate lysis by CD69, and inhibition of lysis by NKp30, NKG2D, and NKp44 (arrows point to adherent DPMSCs). Blocking CD94 receptor did not increase cytolytic activity of NK cells. Experiments were carried out in triplicate and repeated 10 times using NK cells and DPMSCs prepared from the peripheral blood of 10 different healthy donors and 10 different fetal membranes, respectively. Scale bars = 50 μm My answer to AQ3: the lysis was 100% and the correct arrows should be arrowheads point at cell suspenstion as corrected in the text. Modified figure is attached

To identify which NK cell receptors mediated DPMSC lysis by NK cells, we used the monoclonal antibody-mediated masking approach in cytolytic experiments as previously described [13]. NK cells were used against allogenic DPMSCs. Antibody-mediated masking of DNAM and NKp46 did not inhibit NK cell lysis of DPMSCs as there was no sign of intact adherent DPMSCs, cells were ruptured, and cellular debris was obvious in suspension (Fig. 4). In contrast, blocking CD69 resulted in moderate inhibition of NK cell lysis of DPMSCs (~ 50% of DPMSCs were adherent) while NK cell lysis of DPMSCs was significantly inhibited after blocking NKp30, NKG2D, and NKp44 (> 75% of DPMSCs were adherent) (Fig. 4). In addition, blocking CD94/NKG2A (HLA-E specific inhibitory receptor) did not increase NK cell cytolytic activity against DPMSCs (Fig. 4).

### Functional activities of NK cells exposed to DPMSCs

**Fig. 5:**
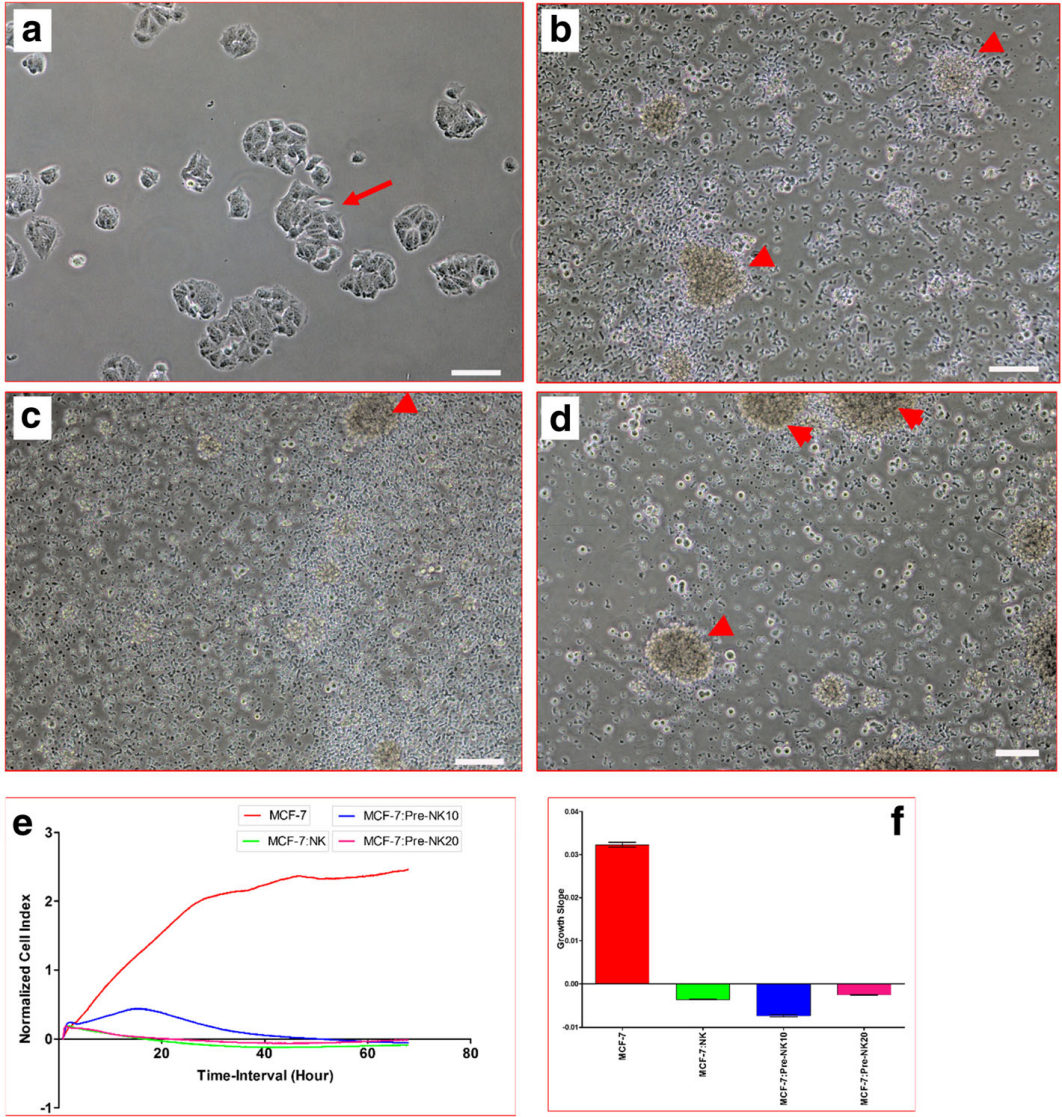
Natural killer (NK) cell interaction with MCF-7 breast cancer cells evaluated by culturing IL-2-activated NK cells with DPMSCs at different NK:DPMSC ratios in a cytolytic experiment. Following 24-h incubation with DPMSCs, NK cells were harvested, purified, and then added to MCF-7 cells at a 1:10 MCF-7:NK ratio, and NK cytolytic activity against MCF-7 cells was then evaluated using microscopic examination and the xCELLigence real-time cell analyzer. Representative phase-contrast microscopic images show a complete lysis of MCF-7 cells (no sign of intact adherent MCF-7 and cells were also ruptured as cellular debris was obvious in suspension (arrowheads)) by untreated NK cells (NK cells were initially activated with 100 U/ml IL-2 for 72 h) (**b**), treated NK cells (IL-2-activated NK cells cocultured with DPMSCs at 10:1 and 20:1 NK:DPMSC ratios) (**c,d**) as compared with MCF-7 cells (arrow) cultured alone (**a**) within 24 h of culture. The results of the xCELLigence showed that after 70 h of culture, MCF-7 were completely lysed by untreated NK cells and treated NK cells (described above) as shown by the cell index (**e**) and growth slope (**f**). The cell index was reduced almost to zero for MCF-7 cocultured with NK cells indicating no sign of intact cells. Experiments were carried out in triplicate and repeated 10 times using NK cells and DPMSCs prepared from the peripheral blood of 10 different healthy donors and 10 different fetal membranes, respectively. Scale bars = 50 μm

To further study the possible modulatory effects of DPMSCs on NK cell cytolytic activity, NK cells were activated with IL-2 for 72 h and then used in the NK/DPMSC cytolytic experiment where NK cells lysed DPMSCs at 10:1 and 20:1 NK:DPMSC ratios. Following 24 h of culture with DPMSCs, NK cells were harvested from the NK/DPMSC cytolytic experiment, purified, and then used to examine their ability to lyse MCF-7 breast cancer cells in a NK/MCF-7 cytolytic experiment at a 1:10 MCF-7:NK cell ratio. This ratio was chosen because it is the lowest ratio that caused a complete lysis of DPMSCs. IL-2-treated NK cells cultured without DPMSCs served as a control. Within 24 h of culture, MCF-7 cells were completely lysed (no sign of intact adherent MCF, cells were ruptured, and cellular debris was obvious in suspension) by DPMSC-untreated NK cells (Fig. 5b) and DPMSC-treated NK cells (Fig. 5c, d) as compared with MCF-7 cells cultured alone (untreated) (Fig. 5a). This NK cell cytolytic activity against MCF-7 cells was also confirmed by the xCELLigence real-time system. The cell index for MCF-7 cocultured with NK cells precultured with DPMSCs or cultured alone was zero, indicating that they were no signs of MCF-7 adhesion or proliferation (cell lysis) (Fig. 5e, f).

### Expression of immune molecules by NK cells exposed to DPMSCs

**Fig. 6:**
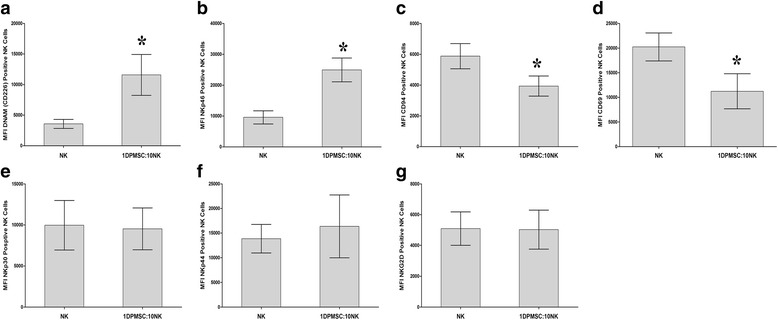
Flow cytometric analysis of the indicated activating and inhibiting receptors by natural killer (NK) cells harvested from NK/DPMSC cytolytic experiments. *Decidua parietalis* mesenchymal stem/stromal cells (DPMSCs) significantly increased NK cell expression of DNAM (**a**) and NKp46 (**b**), while the expression of CD94 (**c**) and CD69 (**d**) was significantly decreased. DPBMSCs had no significant effect on NK cell expression of NKp30 (**e**), NKp44 (**f**), and NKG2D (**g**) (*P* > 0.05). The levels of the expression of activating and inhibitory receptors in NK cells were recorded as median fluorescence intensity (MFI) as determined by flow cytometry. Experiments were carried out in triplicate and repeated 10 times using NK cells and DPMSCs prepared from the peripheral blood of 10 different healthy donors and 10 different fetal membranes, respectively. Bars represent standard errors. *P < 0.05, versus untreated NK cells

To examine whether the phenotype of IL-2-activated NK cells in the NK/DPMSC cytolytic experiment was modified after culturing with DPMSCs at 10:1 NK:DPMSC ratio, several immune markers were assessed by flow cytometry and the expression recorded as mean fluorescence intensity (MFI). This ratio was chosen because it was the lowest ratio that caused a complete lysis of DPMSCs. After 24 h of culture, NK cell expression of DNAM and NKp46 (activating receptors) was significantly increased when compared with untreated NK cells (*P* < 0.05; Fig. 6a, b). In contrast, NK cell expression of activating receptor (CD69) and inhibiting receptor (CD94) was significantly decreased as compared with untreated NK cells (*P* < 0.05; Fig. 6c, d) while NK cell expression of NKp30, NKp44, and NKG2D (activating receptors) was not affected as compared with untreated NK cells (Fig. 6e–g).

**Fig. 7:**
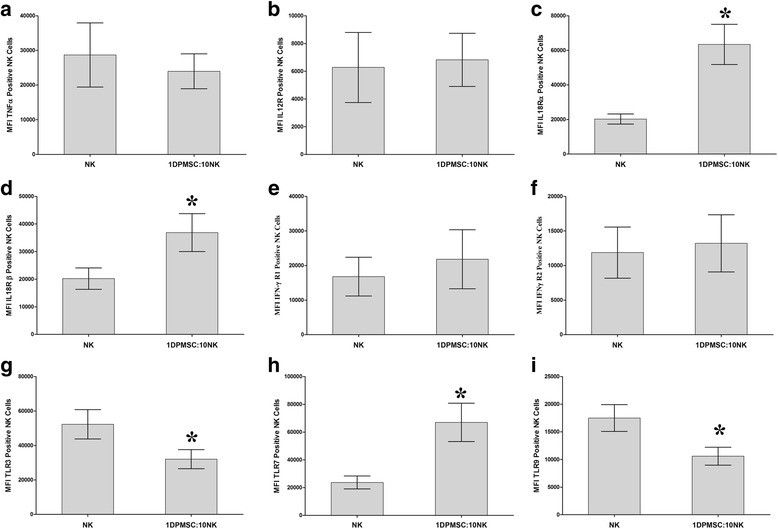
Flow cytometric analysis of the indicated inflammatory molecules by natural killer (NK) cells harvested from NK/DPMSC cytolytic experiments. *Decidua parietalis* mesenchymal stem/stromal cells (DPMSCs) significantly increased NK cell expression of the interleukin-18 receptors IL-18Rα (**c**) and IL-18Rβ (**d**), and Toll-like receptor (TLR)7 (**h**), while the expression of TLR3 (**g**) and TLR (**i**) was significantly decreased. DPMSCs had no significant effect on NK cell expression of tumor necrosis factor (TNF)-α (**a**), IL-12R (**b**), interferon (IFN)-γR1 (**e**), and IFN-γR2 (**f**) (*P* > 0.05). The levels of the expression of activating and inhibitory receptors in NK cells were recorded as median fluorescence intensity (MFI) as determined by flow cytometry. Experiments were carried out in triplicate and repeated 10 times using NK cells and DPMSCs prepared from the peripheral blood of 10 different healthy donors and 10 different fetal membranes, respectively. Bars represent standard errors. *P < 0.05, versus untreated NK cells

Next, we examined whether DPMSCs modulated the NK expression of the immune proteins listed in Table 1. After 24 h of culture with DPMSCs, NK cell expression of IL-18Rα, IL-18Rβ, and TLR7 significantly increased as compared with untreated NK cells (*P* < 0.05; Fig. 7c, d, h). In contrast, NK cell expression of TLR3 and TLR9 significantly decreased as compared with untreated NK cells (*P* < 0.05; Fig. 7g, i), while NK cell expression of TNF-α, IL-12Rβ1, IFN-γR1, and IFN-γR2 was not significantly affected as compared with untreated NK cells (Fig. 7a, b, e, f).

### IL-18 receptor has no role in mediating NK cell cytolytic activity against tumor cells

**Fig. 8:**
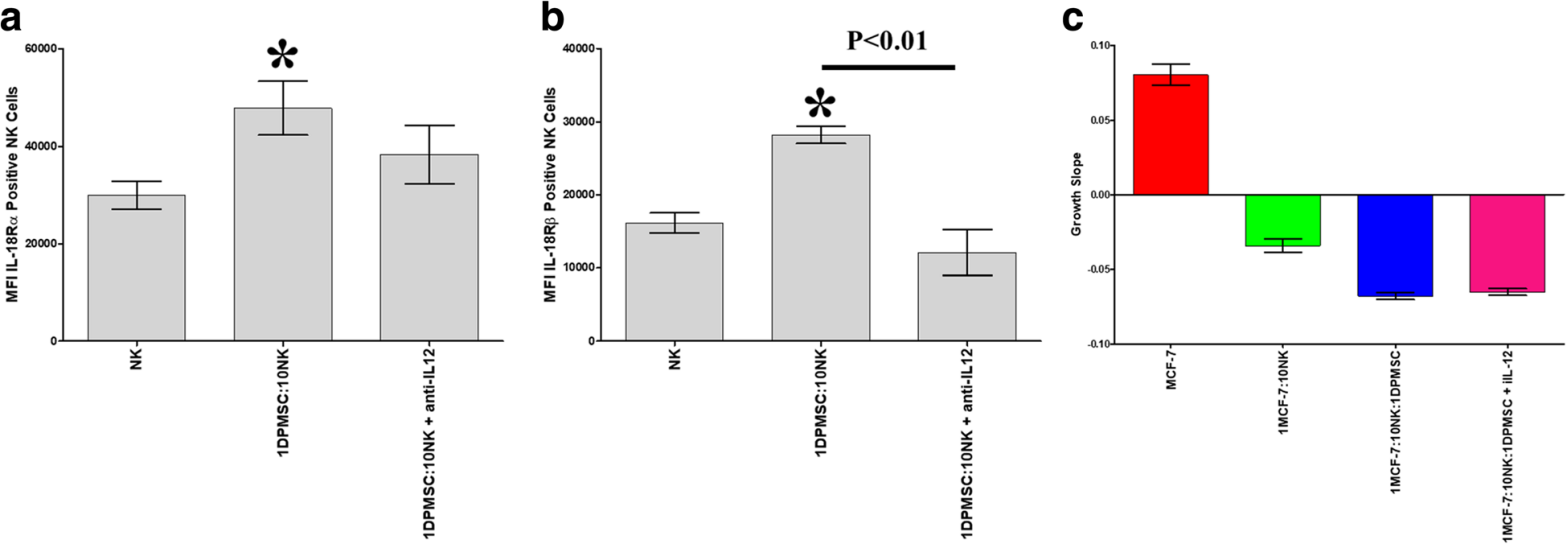
The role of IL-12 in mediating *decidua parietalis* mesenchymal stem/stromal cells (DPMSCs) increasing natural killer (NK) cell expression of interleukin-18 receptor (IL-18R) and the role of IL-18R in mediating NK cell cytolytic activity against MCF-7 breast cancer cells. Inhibiting IL-12 secretion by DPMSCs reduced NK expression of IL-18R as determined by flow cytometry (**a,b**) while NK cell expression of reduced levels of IL-18R did not affect NK cell cytolytic activity against MCF-7 cells as shown by the xCELLigence real-time cell analyzer; MCF-7 were completely lysed by IL-2-activated NK cells cocultured with DPMSCs in the absence or presence of neutralizing IL-12 antibody as compared with MCF-7 cells cultured alone (**c**). Experiments were carried out in triplicate and repeated three times using NK cells and DPMSCs prepared from the peripheral blood of three different healthy donors and three different fetal membranes, respectively. Bars represent standard errors. *P < 0.05

The finding that DPMSCs increased NK cell expression of IL-18Rα and β suggested that IL-18R may mediate NK cytolytic activity against MCF-7 cells. Therefore, we examined the role of IL-18R in mediating NK cell lysis of MCF-7 cells when neutralizing IL-12 antibody was present. We found that inhibiting DPMSC secretion of IL-12 (data not shown) reduced NK cell expression of IL-18Rα and β (Fig. 8a, b). In addition, we also found that NK cell expression of a reduced level of IL-18Rα and β had a comparable cytolytic activity against MCF-7 as compared with NK cells expressing increased levels of IL-18R (Fig. 8c).

## Discussion

In this study, we evaluated the consequences of NK cell interaction with DPMSCs. We found that NK cells preactivated with IL-2 can lyse DPMSCs and this was mediated by different NK receptors interacting with ligands expressed by DPMSCs. In addition, DPMSCs stimulated the proliferation of unactivated NK cells induced to proliferate by IL-2 (Fig. 1a). This even occurred with low numbers of DPMSCs relative to NK cells. For IL-2-activated NK cells, the proliferative ability was also increased, but only with high numbers of DPMSCs relative to NK cells (Fig. 1b). Our results are not consistent with previous studies, which reported that BMMSCs decreased NK cell proliferation [13, 37]. One obvious explanation is that the original microenvironment (i.e., the niche) from which the MSCs were derived plays a role in how MSCs influence NK cell functions. The mechanisms underlying DPMSCs stimulating NK cell proliferation are not known, but it is likely to be mediated by soluble factors produced by DPMSCs. Previously, we reported that DPMSCs produce several cytokines with a proliferative function, such as IL-12, which can stimulate NK cell proliferation [38]. The possible role of IL-12 in DPMSC and NK cell interaction could be addressed in a future study.

A previous study reported that the NK cytolytic activity against BMMSCs is promoted when BMMSCs express low levels of HLA class I molecules [13]. DPMSCs, which express a moderate level of HLA-E and high levels of HLA-ABC molecules, were lysed by NK cells. Our results demonstrate that NK cytolytic activity against DPMSCs is not inhibited by HLA class I molecules. This is consistent with a previous study, which showed that NK cell cytolytic activity is independent of HLA class I molecule expression [39]. Further support for this is our finding that the preconditioning of NK cells with DPMSCs did not inhibit NK cell cytolytic activity against MCF-7 cells, which express HLA-class I antigens (Fig. 5). Our results are different from a previous study, which reported that NK cell cytolytic activity against MCF-7 cells was reduced after the preconditioning of NK cells with BMMSCs [37]. Again, this may reflect the different niches from which the DPMSCs and BMMSCs were derived, influencing their effect on NK cell cytolytic activity.

DPMSCs expressed several ligands specific to activating NK cell receptors (Table 2). As expected, the blockage of NKG2D caused a significant inhibition of NK cytolytic activity against DPMSCs (Fig. 4). However, the blockage of DNAM-1 and NKp46 (i.e., the other NK-activating receptor) did not inhibit NK cell lysis of DPMSCs, even though the expression of these receptors by NK cells was significantly increased by coculture with DPMSCs during the cytolytic experiment (Fig. 4). Thus, these receptors do not appear to play a role in NK cell lysis of DPMSCs, but we cannot preclude the involvement of other NK cell-activating receptors. Indeed, our receptor-blocking experiments showed that NK cell cytolytic activity against DPMSCs was mediated by CD69, NKp30, and NKp44. Importantly, we found that coculture of NK cells with DPMSCs had an inhibitory effect on NK cell expression of CD69, but not NKp30 and NKp44 (Fig. 6). This indicates that DPMSCs may induce the cytolytic activity of NK cells through CD69, but not other receptors (NKp30, NKp44, or NKG2D). However, this will need to be further confirmed in a future study.

The susceptibility of DPMSCs to lysis by NK cells raises the question of the efficacy of DPMSC therapy. DPMSC lysis by NK cells suggests that DPMSCs would be removed shortly after their transplantation into patients. However, this would not necessary rule out their long-term reparative efficacy thorough paracrine mechanisms on other cells or tissues. Our previous work showed that DPMSCs produce many factors, such as IL-10, hepatocyte growth factor (HGF), and transforming growth factor (TGF)-β1 [7]. These factors can modulate the functions of lymphocytes (T, B, and NK cells) and also have reparative properties [11]. Therefore, it is possible these factors could mediate DPMSC modulatory and reparative functions in vivo. However, this should be addressed in a future study.

Resting, nonactivated NK cells did not lyse DPMSCs (Fig. 2b–e). Instead, NK cells decreased DPMSC proliferation in a dose-dependent manner (Fig. 2f, g). Importantly, the exposure of NK cells to IL-2 induced their cytolytic activity against DPMSCs (Fig. 3d–g). These results suggest that the inflammatory environment enhances NK cytolytic activity against DPMSCs. The NK cell inflammatory phenotype is also enhanced by DPMSCs. We found that DPMSCs induced NK cell expression of inflammatory factors (IL-18Rα and β) (Fig. 7). IL-18 receptors mediate the functions of IL-18, an inflammatory cytokine that is essential for NK cell functional activity [40]. IL-18 acts predominantly to stimulate T-helper cell responses through the induction of IFN-γ. IL-18 also enhances NK cell secretion of inflammatory mediators, such as TNF-α and IL-1β [40]. In addition, IL-18 can prime NK cells, which potentiate the stimulatory effects of dendritic cells on tumor- specific T-helper cells and cytotoxic lymphocytes in cancer patients [41]. Therefore, our results suggest that DPMSCs provide a stimulatory signal activating NK cells through the IL-18 pathway, and this in turn induces NK cells to promote T-cell anticancer functions. IL-18R expression in NK cells is induced by IL-12 [42], and therefore we examined whether IL-12 secreted by DPMSCs mediated NK expression of IL-18R. We found that neutralizing IL-12 secretion by DPMSCs reduced NK cell expression of IL-18R indicating that IL-12 mediates DPMSCs increasing NK cell expression of IL-18R. We also found that NK cell expression of reduced levels of IL-18R had no mediatory effect on NK lysing MCF-7. This suggests that IL-18R may mediate NK cytolytic activity against cancer cells through other pathways.

Current anticancer therapeutic approaches against cancer cells is to exploit NK cell cytolytic activity. Different strategies that employ NK cells include in vivo stimulation of NK cell functions by inflammatory cytokines, such as IL-2, NK cell adoptive transfers, and the induction of NK cell antitumor activities by agonists, such as antibodies [43–45]. NK cells express TLRs, such as TLR3, TLR7, and TLR9, which bind to ligands expressed by pathogens [20, 46]. TLRs stimulate the functional activity of NK cells directly or by dendritic cells [20, 21]. Importantly, these receptors have antitumor activities [47, 48]. Recently, it was shown that an agonist against TLR7 can activate NK cells to completely lyse tumor cells [49]. In this study, coculturing NK cells with DPMSCs induced NK cell expression of TLR7 (Fig. 7). This suggests that DPMSCs could be used to prime NK cells to enhance their antitumor functions.

Our data provide new knowledge on the interactions between DPMSCs and NK cells, and suggest that DPMSCs could be used as part of an antitumor therapeutic strategy. NK cells mediate the responses of the immune system; they can eradicate cancer cells and regulate graft rejection.

## Conclusions

Our data suggest that DPMSCs may not be beneficial for transplantation or immunological diseases. However, preconditioning of NK cells with DPMSCs stimulates NK cell proliferation and induces NK expression of inflammatory as well as antitumor molecules, which could be advantageous for cancer therapy.
